# Trends in antibiotic utilization for patients hospitalized with COVID-19 with and without signs of sepsis

**DOI:** 10.1017/ash.2024.366

**Published:** 2024-10-03

**Authors:** Claire N. Shappell, Michael Klompas, Christina Chan, Tom Chen, Chanu Rhee

**Affiliations:** 1 Division of Pulmonary and Critical Care Medicine, Department of Medicine, Brigham and Women’s Hospital, Boston, MA, USA; 2 Department of Population Medicine, Harvard Medical School/Harvard Pilgrim Health Care Institute, Boston, MA, USA; 3 Division of Infectious Diseases, Department of Medicine, Brigham and Women’s Hospital, Boston, MA, USA

## Abstract

**Objective:**

To assess trends in antibiotic prescribing for patients hospitalized with COVID-19 with and without sepsis.

**Design:**

Retrospective cohort study using electronic health record (EHR) data.

**Setting:**

Five hospitals in eastern Massachusetts.

**Patients:**

Adults (≥18 years) hospitalized with community-onset SARS-CoV-2 infections between March 2020 and November 2022.

**Methods:**

We assessed quarterly trends in the use of prolonged initial antibiotic therapy (≥4 antibiotic days within one week of admission, including discharge antibiotics) amongst COVID-19 patients with and without sepsis, defined using clinical signs of organ dysfunction before hospital day 3. Poisson regression models were used to adjust for baseline characteristics and severity of illness.

**Results:**

Of 431,017 hospitalizations in the study period, 21,563 (5.0%) had community-onset COVID-19. 4,769/21,563 (20.5%) presented with sepsis. Prolonged antibiotics were prescribed in 2,323/4,769 (48.7%) COVID-19 patients with sepsis and 2,866/16,794 (17.1%) without sepsis despite low rates of positive bacterial cultures on admission (15.0% vs 6.3%, respectively). Quarterly rates of prolonged antibiotics declined between the first and second pandemic quarters for both sepsis (66.8% to 43.9%) and no-sepsis (31.8% to 24.4%) groups. However, there was no significant change thereafter through November 2022 in either group (quarterly aORs 1.02, 95% CI 0.99–1.05 and 1.01, 95% CI 0.99–1.03, respectively).

**Conclusions:**

Prolonged antibiotics were common in hospitalized COVID-19 patients with and without sepsis during the first 33 months of the pandemic despite low rates of proven bacterial infection. Decreases in antibiotic utilization occurred primarily between the first and second pandemic quarter with no further reduction thereafter.

## Introduction

Sepsis, the syndrome of life-threatening organ dysfunction caused by a dysregulated host response to infection, is most commonly caused by bacterial infections but can be triggered by any type of pathogen.^1–3^ The COVID-19 pandemic has raised awareness that viruses can cause sepsis even in the absence of bacterial co-infection and that viral sepsis is associated with similar or higher mortality rates than bacterial sepsis.^4–6^ However, many clinicians administer empiric antibiotics to patients with suspected sepsis even if a viral pathogen is identified,^7^ despite lack of benefit and potential for harm in patients with non-bacterial sepsis. Although prior studies have reported high rates of empiric antibiotic use in patients hospitalized with community-onset COVID-19 early in the pandemic, and some have indicated that antibiotic utilization rates have decreased over time,^8–10^ antibacterial prescribing in patients with SARS-CoV-2 presenting with sepsis has not been described. We aimed to estimate the prevalence and trends of antibiotic use for patients hospitalized with COVID-19 with and without sepsis across the first 33 months of the pandemic.

## Materials and methods

### Study setting, population, and design

We performed a retrospective study using electronic health record (EHR) data from five Massachusetts hospitals in the Mass General Brigham (MGB) healthcare system. We included all patients ≥18 years old who were admitted, placed under observation, or died in the Emergency Department (ED) between March 1, 2020, and November 30, 2022, with community-onset COVID, defined as a positive SARS-CoV-2 PCR or application of an institutional COVID-19 “flag” in the EHR between 3 days prior to hospitalization and hospital day 2 (with hospital day 1 being the day of admission). Patients who also had a positive COVID-19 PCR or flag 30–90 days prior to admission were excluded to minimize false positives due to recent infections.^11^

Community-onset SARS-CoV-2-associated sepsis was defined using previously validated EHR-based criteria adapted from CDC’s Adult Sepsis Event definition (positive PCR or flag and ≥1 acute organ dysfunction on hospital day ≤2 including oxygen support above nasal cannula, vasopressors, elevated lactate, or changes in baseline creatinine, bilirubin, or platelets).^6^ This definition was previously found to perform well identifying sepsis due solely or primarily to SARS-CoV-2 infection (90.6% sensitivity, 91.2% specificity).^6^

### Outcomes and analysis

The primary outcome of interest was rate of prolonged early antibiotic therapy, defined as ≥4 antibiotic days within the first week following admission (including antibiotics prescribed on discharge if discharged before hospital day 7). We used this definition to focus on patients treated with antibiotics beyond standard 48–72 hour empiric treatment windows. Secondarily, we examined rates of initial antibiotics, defined as any antibiotic administration (ie, ≥1 dose) on hospital days 0–2. Please see Supplement Table 1 for list of included antibiotics.

We calculated quarterly rates of prolonged antibiotic courses in community-onset COVID patients with and without sepsis and analyzed trends using Poisson generalized linear models. We estimated quarterly odds for receipt of prolonged antibiotics relative to the first pandemic quarter using logistic regression to adjust baseline characteristics and severity of illness, including age, gender, race, body mass index (BMI), select Elixhauser comorbidities, worst vital signs within 24 hours of arrival (maximum heart rate, respiratory rate, and temperature; minimum systolic blood pressure), maximum lactate within 24 hours, maximum oxygen support device within 24 hours, and blood, sputum, or urine cultures collected on hospital days 0–2 positive for potentially pathogenic organisms. Cultures positive for common commensals were excluded. Urine cultures required >100,000 CFU/ml to be considered potentially pathogenic. Sensitivity analyses were performed excluding the first study quarter given that COVID-19 care evolved dramatically within the first few months of the pandemic. In addition, we calculated quarterly rates of bacterial cultures (blood, sputum, and urine) obtained during hospital days −1 to 2 and analyzed crude and adjusted trends using Poisson generalized linear models as above.

Statistical significance was established for p-values less than 0.05. Data preparation was performed using SAS version 9.4 (2016, Cary, NC) and Stata version 17 (2021, College Station, TX), while all statistical analyses were done using R version 4.1.3 (2022, Vienna, Austria). The study was approved by the Mass General Brigham Institutional Review Board (protocol 2020P001631; approved with a waiver of consent due to minimal risk of harm).

## Results

Of 431,017 hospitalizations in the study period, 21,563 (5.0%) patients presented with community-onset COVID-19, of whom 4,769 (22.1%) met criteria for community-onset sepsis. Characteristics of COVID-19 patients with and without sepsis are shown in Table 1. Crude in-hospital mortality was 23.3% for COVID-19 patients with signs of sepsis vs 2.5% for those without signs of sepsis. 3,197/4,769 (67.0%) COVID-19 patients with sepsis had at least one clinical culture obtained on hospital days 0–2 and 715/4,769 (15.0%) had at least one culture positive for a potentially pathogenic organism. In patients without sepsis, 6,045/16,794 (36.0%) had any culture obtained and 1,053/16,794 (6.3%) had a positive culture (see Table 2 for list of most common positive culture sites and organisms).

**Table 1.** Characteristics, outcomes, and antibacterials prescribing for patients hospitalized with community-onset COVID-19 with and without sepsis

Patient/Encounter Characteristics	Community-Onset COVID	
	With Sepsis	Without Sepsis
	n = 4,769	n = 16,794
Age, median [IQR], y	66 [54, 78]	62 [44, 76]
Male, n (%)	2,748 (57.6)	8,094 (48.2)
BMI, median [IQR]	28.0 [24.0, 33.3]	27.6 [23.8, 32.4]
Elixhauser Mortality Score, median [IQR]	6 [−2, 20]	0 [−4, 12]
Location prior to admission, n (%)		
Home/Outpatient	3,660 (76.8)	15,202 (90.5)
Transfer from acute-care hospital	854 (17.9)	1,128 (6.7)
Transfer from subacute or long-term care facility	222 (4.7)	347 (2.1)
Missing or Other*	33 (0.7)	117 (0.7)
Hospital LOS, median [IQR], d	9 [5, 18]	4 [2, 7]
Required ICU admission, n (%)	2,669 (56.0)	1,003 (6.0)
Any culture obtained HD −1 to 2	3,197 (67.0)	6,045 (36.0)
Any blood culture HD −1 to 2	2,910 (61.0)	4,832 (28.8)
Any sputum culture HD −1 to 2	1,061 (22.3)	560 (3.3)
Any urine culture HD −1 to 2	1,223 (25.6)	2,479 (14.5)
Any positive culture HD 0–2, n (%)	715 (15.0)	1,053 (6.3)
Positive blood culture HD 0–2, n (%)	156 (3.3)	169 (1.0)
Positive sputum culture HD 0–2, n (%)	355 (7.4)	131 (0.8)
Positive urine culture HD 0–2, n (%)	296 (6.2)	808 (4.8)
Discharge disposition, n (%)		
Home	2,205 (46.2)	13,724 (81.7)
In-hospital death	1,111 (23.3)	415 (2.5)
Hospice	110 (2.3)	211 (1.3)
Non-acute care facility	1,334 (28.0)	2,373 (14.1)
Acute hospital transfer (non-MGB)	9 (0.2)	71 (0.4)
Any initial (<HD3) antibiotics, n (%)	3,204 (67.2)	5,517 (32.9)
Prolonged early antibiotics (≥4 d/week), n (%)	2,375 (49.8)	3,435 (20.5)
Days of antibiotics within 1 week of admission including discharge antibiotics for ALL patients, median (IQR)	3 [1, 6]	0 [0, 2]
Days of antibiotics within 1 week of admission including discharge antibiotics for patients receiving PROLONGED antibiotics, median (IQR)	6 [5–7]	6 [5–7]
Common Initial Antibiotics Classes Received (HD 0–2)		
Anti-Pseudomonal Beta-Lactams	1,547 (32.4)	1,311 (7.8)
Anti-MRSA	1,474 (30.9)	1,148 (6.8)
Macrolide	1,140 (23.9)	1,396 (8.3)
Respiratory Fluoroquinolone	77 (1.6)	139 (0.8)
Common Initial Specific Antibiotics Received (HD 0–2)		
Ceftriaxone	1,594 (33.4)	2,577 (15.3)
Vancomycin	1,440 (30.2)	1,092 (6.5)
Cefepime	1,231 (25.8)	887 (5.3)
Azithromycin	1,140 (23.9)	1,396 (8.3)
Doxycycline	627 (13.1)	863 (5.1)
Piperacillin-Tazobactam	274 (5.7)	359 (2.1)
Cefazolin	171 (3.6)	240 (1.4)

Note. *Other includes court/law enforcement (n = 20), ambulatory surgery center (n = 10), hospice (n = 1), missing or not available (n = 119).

**Table 2.** Site and organisms associated with positive cultures obtained before hospital day 3 in patients with community-onset COVID-19

Culture/Micro-organism Category	Positive Culture Site			
	Blood	Sputum	Urine	Any
	n (%)	n (%)	n (%)	n (%)
Positive Culture Site	325/21,563 (1.5)	486/21,563 (2.3)	1,104/21,563 (5.1)	1,768/21,563 (8.2)
Organism				
*Escherichia* spp.	90 (27.7)	40 (8.2)	579 (52.5)	635 (35.9)
*Staphylococcus aureus*	77 (23.7)	223 (45.9)	58 (5.3)	307 (1.4)
*Klebsiella* spp.	34 (10.5)	54 (11.1)	188 (17.0)	254 (14.4)
*Enterococcus* spp.	26 (8.0)	8 (1.7)	165 (15.0)	185 (10.5)
*Streptococcus* spp.	71 (21.9)	55 (11.3)	23 (2.1)	131 (7.4)
*Pseudomonas* spp.	14 (4.3)	55 (11.3)	68 (6.2)	121 (6.8)
*Proteus* spp.	23 (7.1)	12 (2.5)	93 (8.4)	109 (6.2)
Gram-negative NOS	6 (1.9)	28 (5.8)	11 (1)	41 (2.3)
*Enterobacte*r spp.	6 (1.9)	11 (2.3)	20 (1.8)	29 (1.6)
*Haemophilus* spp.	1 (0.3)	28 (5.8)	0	29 (1.6)
*Citrobacter* spp.	2 (0.6)	3 (0.6)	24 (2.2)	26 (1.5)
*Serratia* spp.	4 (1.2)	15 (3.1)	8 (0.7)	20 (1.1)
*Stenotrophomona*s spp.	2 (0.6)	13 (2.7)	1 (0.1)	15 (0.9)
*Staphylococcus lugdunensis*	8 (2.5)	1 (0.2)	4 (0.4)	11 (0.6)

3,204/4,769 (67.2%) patients with community-onset COVID-19 with signs of sepsis received ≥1 antibiotic before hospital day 3 and 2,375/4,769 (49.8%) received ≥4 days of antibiotics within the first week (“prolonged antibiotics”), including 1,817/4,054 (44.8%) of patients without positive cultures before hospital day 3. The median duration of antibiotics during the first week among those who received prolonged antibiotics was 6 days (IQR 5–7). Ceftriaxone (n = 1,594, 33.4%), vancomycin (n = 1,440, 30.2%), and cefepime (n = 1,231, 25.8%) were the most common antibiotics administered (Table 1). By contrast, 3,435/16,794 (20.5%) patients with community-onset COVID-19 without sepsis received prolonged antibiotics. 1,699/4,769 (35.6%) of COVID-19 patients with sepsis and 12,408/16,794 (73.9%) without sepsis were discharged in less than 7 days. Of those discharged in less than 7 days, 133/1,699 (7.8%) with sepsis and 1,058/12,408 (8.5%) without sepsis were prescribed antibiotics on discharge.

**Figure 1.**
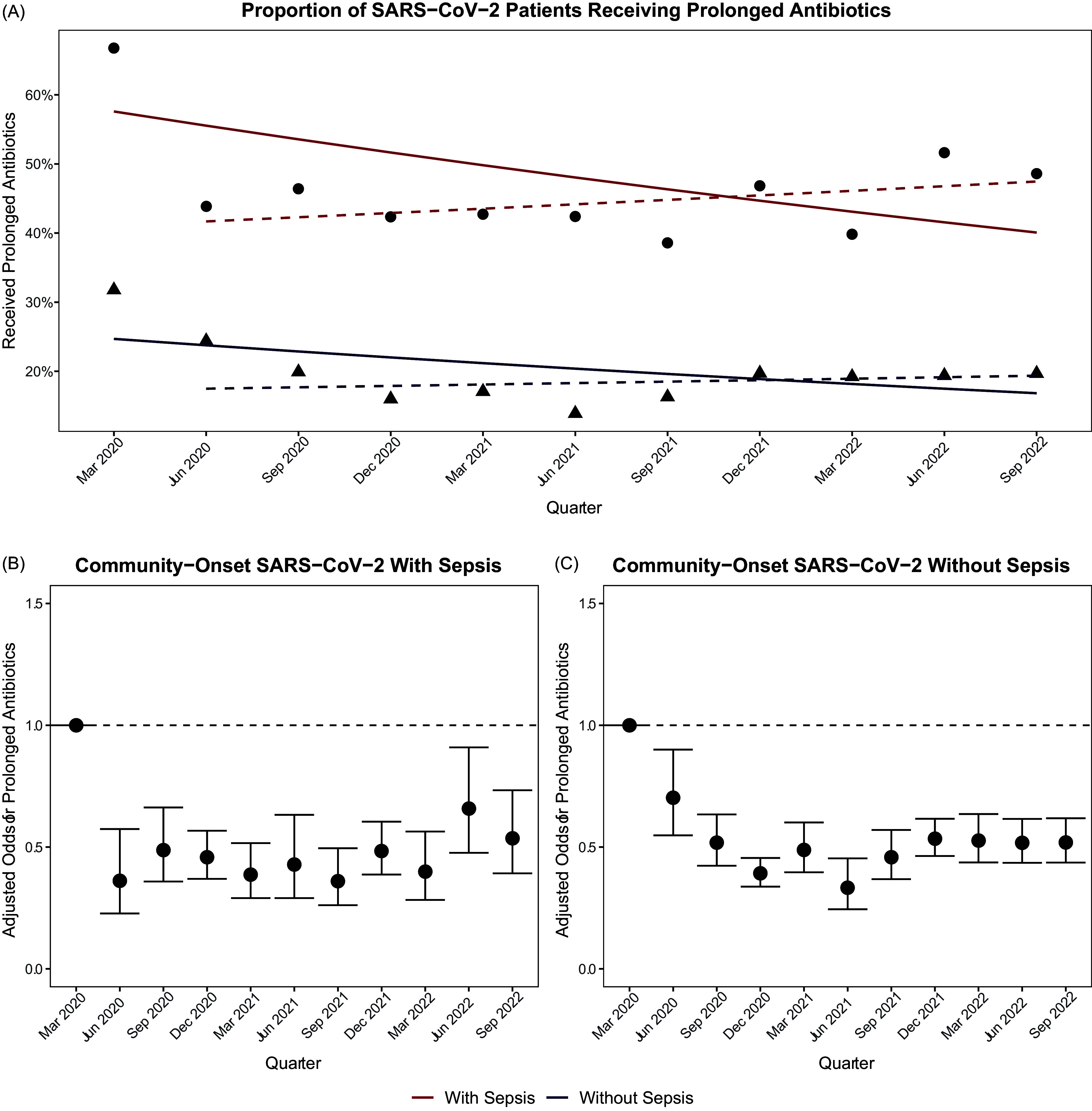
Crude (A) and adjusted (B and C) rates of prolonged initial antibiotics courses in hospitalized COVID-19 patients presenting with vs without signs of sepsis. Dotted lines in (A) represent trends when quarter 1 is excluded from analysis.

Crude rates of prolonged antibiotic use for COVID-19 patients with sepsis decreased from 66.8% in the first quarter to 48.6% in the last quarter (IRR 0.96/quarter, 95% CI 0.95–0.98); results were similar when adjusting for baseline characteristics and severity-of-illness (quarterly aOR 0.95, 95% CI 0.93–0.97). When the first quarter was excluded from the analysis, however, there was no change over time (IRR 1.02/quarter, 95% CI 0.99–1.03, aOR 1.02, 95% CI 0.99–1.05). For COVID without sepsis, crude rates of prolonged antibiotics decreased from 31.8% to 19.7% (IRR 0.96/quarter, 95% CI 0.95–0.97, aOR 0.96, 95% CI 0.94–0.97), but this was also no longer significant when the first quarter was excluded (IRR 1.01/quarter, 95% CI 0.99–1.03, aOR 1.01, 95% CI 0.99–1.03) (Figure 1, see Supplement for full model results). Similar findings were noted for any initial antibiotic therapy (Supplement). Rates of bacterial testing on admission decreased modestly over the study period in crude and adjusted analysis for COVID-19 patients with sepsis (IRR 0.97/quarter, 95% CI 0.96–0.98, aOR 0.97, 95% CI 0.94–0.99) and without sepsis (IRR 0.98/quarter, 95% CI 0.97–0.98, aOR 0.98, 95% CI 0.97–0.996); however, trends were not significant when the first quarter was excluded (Supplement).

## Discussion

In this retrospective study of over 21,000 patients hospitalized with COVID-19 during the first 33 months of the pandemic, antibiotics were given to more than two-thirds of COVID-19 patients admitted with signs of sepsis and one third of patients without sepsis. Most antibiotic courses in both groups were ≥4 days despite low rates of documented bacterial infection. In crude and adjusted analysis, antibiotic use decreased for patients with and without sepsis between the first and last quarters of the analysis. Following the first pandemic quarter, however, prolonged antibiotic use remained high and stable for the rest of the study period for both community-onset COVID-19 with sepsis (44.5%) and without sepsis (18.5%). Similarly, there was a significant reduction in antibiotic starts (of any duration) between the first and second quarter of the pandemic but no change thereafter.

Our results are consistent with other studies documenting reductions in antibiotic use for COVID-19 patients^8–10^ but clarify that decreases occurred early in the pandemic and that usage has remained elevated and stable since then. High ongoing rates of antibacterial prescribing for patients with COVID-19 likely reflect clinicians’ concern for possible bacterial co-infection, particularly amongst patients with signs of sepsis. However, rates of early microbiologic culture decreased modestly during the study period, driven primarily by a reduction following pandemic quarter 1. Only 15% of patients with sepsis and 6% of patients without sepsis had early positive bacterial cultures. This suggests ample opportunity to improve antimicrobial stewardship and reduce risks of antibiotics-associated harms through more attention to obtaining high quality cultures as well as rapid antibiotic de-escalation, even in patients who meet sepsis criteria.

Our study has several limitations. Our analysis was restricted to a single regional healthcare system which may limit generalizability. Identifying sepsis due to COVID-19 is challenging because attributing organ dysfunction to infection can be nuanced. We used a previously-validated approach to identify sepsis using objective clinical data to mitigate bias related to changes in documentation or coding during the study period. In addition, some COVID-19 patients treated with antibiotics potentially had bacterial infections that were never cultured (e.g. sputum specimens not sent) and may therefore have been appropriately treated with antibiotics. Thus, our results should not be taken to imply that all antibiotics for community-onset COVID-19 infection with sepsis represent overtreatment; further study is needed to identify the optimal approach to antibiotic use in this group. Finally, our study does not address whether prolonged antibiotics in this population were associated with adverse effects. Previous studies, however, have found increased risk of mortality and *Clostridioides difficile* infections in patients with community-onset culture-positive sepsis who received unnecessarily broad therapy;^12^ future studies are needed to assess whether and what harms may be associated with empiric antibiotics in patients with viral sepsis.

In conclusion, prolonged empiric antibiotics were commonly administered to patients hospitalized with community-onset COVID-19, particularly in those with signs of sepsis, during the first 33 months of the pandemic. While the prolonged use of antibiotics decreased for hospitalized COVID-19 patients with and without sepsis between the first and second quarters, prescribing rates were stable thereafter and substantially higher than the rate of proven bacterial coinfection. Our findings highlight an ongoing opportunity to improve antibiotic use in patients presenting with severe respiratory viral infections.

## Supporting information

Shappell et al. supplementary material Shappell et al. supplementary material

## Supplementary material

For supplementary material accompanying this paper visit http://doi.org/10.1017/ash.2024.366. click here to view supplementary material
