# Supplementary material for: Trends in antibiotic utilization for patients hospitalized with COVID-19 with and without signs of sepsis
Source: Antimicrob Steward Healthc Epidemiol. 2024 Oct 3;4(1):e143. doi: 10.1017/ash.2024.366 (PMC11450668; doi:10.1017/ash.2024.366)

**ONLINE SUPPLEMENT**

**Table of Contents**

1. Supplement Table 1: List of included antibiotics
2. Supplement Table 2. Full adjusted model results for trends in prolonged antibiotics
3. Supplement Table 3. Summary of model results for receipt of initial antibiotics
4. Supplement Figure 1. Adjusted Rates of Initial Antibiotics in Hospitalized COVID-19 Patients Presenting With (A) vs. Without (B) Signs of Sepsis
5. Supplement Figure 2. Crude and Adjusted Rates of Early (hospital day -1 to 2) Microbiologic Culture including Blood, Sputum, or Urine in Hospitalized COVID-19 Patients Presenting With vs Without Signs of Sepsis

**Supplement Table 1. List of included antibiotic medications**

| **ANTIBIOTIC NAME** | **ROUTE** |
| --- | --- |
| AMIKACIN | IV |
| AMOXICILLIN | PO |
| AMOXICILLIN_CLAVULANATE | PO |
| AMPICILLIN | IM |
| AMPICILLIN | IV |
| AMPICILLIN | PO |
| AMPICILLIN_SULBACTAM | IV |
| AZITHROMYCIN | IV |
| AZITHROMYCIN | PO |
| AZTREONAM | IV |
| BACITRACIN | IM |
| CEFACLOR | PO |
| CEFADROXIL | PO |
| CEFAZOLIN | IV |
| CEFDINIR | PO |
| CEFEPIME | IM |
| CEFEPIME | IV |
| CEFIDEROCOL | IV |
| CEFIXIME | PO |
| CEFOTAXIME | IV |
| CEFOTETAN | IV |
| CEFOXITIN | IM |
| CEFOXITIN | IV |
| CEFPODOXIME | PO |
| CEFTAROLINE | IV |
| CEFTAZIDIME | IM |
| CEFTAZIDIME | IV |
| CEFTAZIDIME_AVIBACTAM | IV |
| CEFTOLOZANE_TAZOBACTAM | IV |
| CEFTRIAXONE | IM |
| CEFTRIAXONE | IV |
| CEFUROXIME | IV |
| CEFUROXIME | PO |
| CEPHALEXIN | PO |
| CIPROFLOXACIN | IV |
| CIPROFLOXACIN | PO |
| CLARITHROMYCIN | PO |
| CLINDAMYCIN | IM |
| CLINDAMYCIN | IV |
| CLINDAMYCIN | PO |
| DALBAVANCIN | IV |
| DAPTOMYCIN | IV |
| DELAFLOXACIN | PO |
| DEMECLOCYCLINE | PO |
| DICLOXACILLIN | PO |
| DOXYCYCLINE | IV |
| DOXYCYCLINE | PO |
| ERAVACYCLINE | IV |
| ERTAPENEM | IM |
| ERTAPENEM | IV |
| FIDAXOMICIN | PO |
| FOSFOMYCIN | PO |
| GENTAMICIN | IM |
| GENTAMICIN | IV |
| IMIPENEM | IV |
| IMIPENEM_RELEBACTAM | IV |
| LEFAMULIN | PO |
| LEVOFLOXACIN | IV |
| LEVOFLOXACIN | PO |
| LINEZOLID | IV |
| LINEZOLID | PO |
| MEROPENEM | IV |
| MEROPENEM_VABORBACTAM | IV |
| METRONIDAZOLE | IV |
| METRONIDAZOLE | PO |
| MINOCYCLINE | IV |
| MINOCYCLINE | PO |
| MOXIFLOXACIN | IV |
| MOXIFLOXACIN | PO |
| NAFCILLIN | IV |
| NEOMYCIN_POLYMYXIN | PO |
| NITROFURANTOIN | PO |
| OMADACYCLINE | IV |
| OMADACYCLINE | PO |
| ORITAVANCIN | IV |
| OXACILLIN | IV |
| PENICILLIN |  |
| PENICILLIN | IM |
| PENICILLIN | IV |
| PENICILLIN | PO |
| PIPERACILLIN_TAZOBACTAM | IV |
| POLYMYXIN_B | IV |
| POLYMYXIN_B | PO |
| STREPTOMYCIN | IV |
| TEDIZOLID | IV |
| TEDIZOLID | PO |
| TELAVANCIN | IV |
| TETRACYCLINE | PO |
| TIGECYCLINE | IV |
| TOBRAMYCIN | IV |
| TRIMETHOPRIM | PO |
| TRIMETHOPRIM_SULFAMETHOXAZOLE | IV |
| TRIMETHOPRIM_SULFAMETHOXAZOLE | PO |
| VANCOMYCIN | IV |
| VANCOMYCIN | PO |

**Supplement Table 2. Full adjusted model results for trends in prolonged antibiotics**

**2a. Adjusted analyses treating quarter as a continuous variable, COVID-19 with Sepsis**

|  | **Prolonged Antibiotics** | | |
| --- | --- | --- | --- |
| *Predictors* | *Odds Ratios* | *95% CI* | *p* |
| (Intercept) | 0.196 | 0.052 – 0.643 | **0.010** |
| Quarter [categorical, Ref=1] | 0.947 | 0.926 – 0.968 | **<0.001** |
| Encounter age [continuous] | 0.999 | 0.994 – 1.004 | 0.649 |
| sex [Male] [binary, Ref=female] | 1.093 | 0.955 – 1.253 | 0.197 |
| Hispanic, all races [categorical, Ref=Non-Hispanic White] | 0.921 | 0.757 – 1.119 | 0.407 |
| Non-Hispanic Black | 1.081 | 0.865 – 1.351 | 0.494 |
| Non-Hispanic Other, including Asian and Multi-racial | 0.749 | 0.573 – 0.977 | **0.034** |
| Hospital Site 2 [categorical, Ref= Hospital Site 1] | 0.949 | 0.728 – 1.236 | 0.698 |
| Hospital Site 3 | 0.962 | 0.809 – 1.144 | 0.662 |
| Hospital Site 4 | 0.692 | 0.553 – 0.865 | **0.001** |
| Hospital Site 5 | 0.739 | 0.600 – 0.908 | **0.004** |
| insurancetype [Medicaid] [categorical, Ref=private] | 1.095 | 0.884 – 1.357 | 0.404 |
| insurancetype [Medicare] | 0.984 | 0.842 – 1.150 | 0.840 |
| BMI <18.5 [categorical, Ref=BMI 18.5-24.9] | 1.510 | 1.057 – 2.166 | **0.024** |
| BMI >=25 & <30 | 0.988 | 0.828 – 1.180 | 0.896 |
| BMI>=30 & <35 | 0.949 | 0.774 – 1.162 | 0.609 |
| BMI >=35 | 0.859 | 0.699 – 1.054 | 0.146 |
| Elixhauser index mortality [continuous] | 1.007 | 1.002 – 1.012 | **0.010** |
| Elixhauser cancer [binary] | 1.266 | 1.013 – 1.583 | **0.038** |
| Elixhauser diabetes [binary] | 1.168 | 1.010 – 1.351 | **0.036** |
| Elixhauser lung chronic [binary] | 1.173 | 1.010 – 1.363 | **0.037** |
| Elixhauser heart failure [binary] | 1.067 | 0.888 – 1.283 | 0.486 |
| Elixhauser hypertension [binary] | 0.947 | 0.801 – 1.121 | 0.528 |
| Elixhauser renal failure [binary] | 0.942 | 0.796 – 1.115 | 0.487 |
| Elixhauser liver disease [binary] | 1.326 | 1.047 – 1.683 | **0.020** |
| Max 24 Hour temp >=96.8 & <100.4 [categorical, REF= <96.8] | 4.056 | 1.531 – 12.847 | **0.009** |
| Max 24 Hour temp >=100.4 | 6.720 | 2.521 – 21.383 | **<0.001** |
| Max 24 Hour Heart Rate >=91 & <121 [categorical, REF= >=41 &<91] | 1.214 | 1.019 – 1.449 | **0.030** |
| Max 24 Hour Heart Rate >=121 | 1.184 | 0.960 – 1.462 | 0.115 |
| Max 24 Hour Respiratory Rate >=21 & <31 [categorical, REF=>=11 & <21] | 1.320 | 1.039 – 1.681 | **0.024** |
| Max 24 Hour Respiratory Rate >=31 | 1.322 | 1.026 – 1.706 | **0.032** |
| Max 24 Hour lactate >=2 & <3 [categorical, REF= <2] | 0.806 | 0.668 – 0.972 | **0.024** |
| Max 24 Hour lactate >=3 & <4 | 0.950 | 0.722 – 1.250 | 0.711 |
| Max 24 Hour lactate cat >=4 | 0.649 | 0.506 – 0.832 | **0.001** |
| Max 24 Hour lactate missing/lactate not obtained | 0.554 | 0.465 – 0.661 | **<0.001** |
| Max 24 Hour Oxygen Device: nasal cannula [categorical, REF= none] | 1.199 | 0.711 – 2.073 | 0.504 |
| Max 24 Hour Oxygen Device: simple mask | 1.250 | 0.724 – 2.210 | 0.431 |
| Max 24 Hour Oxygen Device: oxymizer | 1.244 | 0.627 – 2.491 | 0.534 |
| Max 24 Hour Oxygen Device: advanced mask | 0.898 | 0.523 – 1.578 | 0.701 |
| Max 24 Hour Oxygen Device: high flow nasal canula | 1.641 | 0.944 – 2.918 | 0.084 |
| Max 24 Hour Oxygen Device: BIPAP | 1.311 | 0.630 – 2.745 | 0.470 |
| Max 24 Hour Oxygen Device: ventilator | 2.005 | 1.174 – 3.510 | **0.012** |
| Max 24 Hour Oxygen Device: ECMO | 181513.566 | 0.000 – NA | 0.951 |
| Min 24 Hour Systolic Blood Pressure >=90 & <121 [categorical, REF=<=89] | 0.644 | 0.547 – 0.757 | **<0.001** |
| Min 24 Hour Systolic Blood Pressure >=121 & <161 | 0.507 | 0.397 – 0.648 | **<0.001** |
| Min 24 Hour Systolic Blood Pressure cat >=161 | 0.585 | 0.114 – 2.453 | 0.478 |
| positive culture (any site) before HD 3 [binary] | 3.569 | 2.892 – 4.427 | **<0.001** |
| Observations | 4374 | | |
| R^2^ Tjur | 0.171 | | |

**2b. Adjusted analyses treating quarter as a continuous variable, COVID-19 without Sepsis**

|  | **Prolonged Antibiotics** | | |
| --- | --- | --- | --- |
| *Predictors* | *Odds Ratios* | *95% CI* | *p* |
| (Intercept) | 0.176 | 0.040 – 0.536 | **0.007** |
| Quarter [categorical, Ref=1] | 0.957 | 0.944 – 0.970 | **<0.001** |
| Encounter age [continuous] | 0.998 | 0.995 – 1.002 | 0.314 |
| sex [Male] [binary, Ref=female] | 1.299 | 1.191 – 1.417 | **<0.001** |
| Hispanic, all races [categorical, Ref=Non-Hispanic White] | 0.751 | 0.661 – 0.851 | **<0.001** |
| Non-Hispanic Black | 0.785 | 0.679 – 0.904 | **0.001** |
| Non-Hispanic Other, including Asian and Multi-racial | 0.657 | 0.546 – 0.787 | **<0.001** |
| Hospital Site 2 [categorical, Ref= Hospital Site 1] | 0.960 | 0.822 – 1.119 | 0.605 |
| Hospital Site 3 | 1.073 | 0.954 – 1.207 | 0.236 |
| Hospital Site 4 | 0.771 | 0.668 – 0.889 | **<0.001** |
| Hospital Site 5 | 1.067 | 0.944 – 1.205 | 0.297 |
| insurancetype [Medicaid] [categorical, Ref=private] | 1.053 | 0.923 – 1.200 | 0.443 |
| insurancetype [Medicare] | 1.006 | 0.907 – 1.116 | 0.904 |
| BMI <18.5 [categorical, Ref=BMI 18.5-24.9] | 1.305 | 1.035 – 1.639 | **0.023** |
| BMI >=25 & <30 | 0.827 | 0.740 – 0.923 | **0.001** |
| BMI>=30 & <35 | 0.745 | 0.652 – 0.849 | **<0.001** |
| BMI >=35 | 0.683 | 0.600 – 0.777 | **<0.001** |
| Elixhauser index mortality [continuous] | 1.005 | 1.001 – 1.008 | **0.011** |
| Elixhauser cancer [binary] | 1.709 | 1.481 – 1.971 | **<0.001** |
| Elixhauser diabetes [binary] | 1.137 | 1.029 – 1.256 | **0.012** |
| Elixhauser lung chronic [binary] | 1.244 | 1.125 – 1.375 | **<0.001** |
| Elixhauser heart failure [binary] | 1.011 | 0.881 – 1.159 | 0.876 |
| Elixhauser hypertension [binary] | 0.961 | 0.862 – 1.072 | 0.475 |
| Elixhauser renal failure [binary] | 1.067 | 0.949 – 1.199 | 0.276 |
| Elixhauser liver disease [binary] | 1.319 | 1.130 – 1.536 | **<0.001** |
| Max 24 Hour temp >=96.8 & <100.4 [categorical, REF= <96.8] | 1.926 | 0.657 – 8.350 | 0.297 |
| Max 24 Hour temp >=100.4 | 3.318 | 1.128 – 14.409 | 0.057 |
| Max 24 Hour Heart Rate >=91 & <121 [categorical, REF= >=41 &<91] | 1.060 | 0.962 – 1.169 | 0.239 |
| Max 24 Hour Heart Rate >=121 | 0.993 | 0.855 – 1.150 | 0.921 |
| Max 24 Hour Respiratory Rate >=21 & <31 [categorical, REF=>=11 & <21] | 0.957 | 0.866 – 1.057 | 0.386 |
| Max 24 Hour Respiratory Rate >=31 | 1.138 | 0.988 – 1.310 | 0.072 |
| Max 24 Hour lactate >=2 & <3 [categorical, REF= <2] | 1.148 | 0.945 – 1.390 | 0.162 |
| Max 24 Hour lactate >=3 & <4 | 1.258 | 0.918 – 1.710 | 0.148 |
| Max 24 Hour lactate cat >=4 | 1.371 | 0.970 – 1.922 | 0.070 |
| Max 24 Hour lactate missing/lactate not obtained | 0.533 | 0.485 – 0.585 | **<0.001** |
| Max 24 Hour Oxygen Device: nasal cannula [categorical, REF= none] | 1.151 | 1.045 – 1.269 | **0.004** |
| Max 24 Hour Oxygen Device: simple mask | 3.588 | 1.221 – 11.345 | **0.022** |
| Max 24 Hour Oxygen Device: oxymizer | 2.558 | 0.100 – 65.148 | 0.508 |
| Max 24 Hour Oxygen Device: advanced mask | 3.946 | 0.372 – 85.749 | 0.266 |
| Max 24 Hour Oxygen Device: high flow nasal canula | 1.002 | 0.049 – 7.394 | 0.999 |
| Max 24 Hour Oxygen Device: BIPAP | 3.320 | 0.114 – 96.406 | 0.431 |
| Max 24 Hour Oxygen Device: ventilator | 0.000 | NA – 4143737.725 | 0.939 |
| Min 24 Hour Systolic Blood Pressure cat [2] | 1.018 | 0.876 – 1.184 | 0.820 |
| Min 24 Hour Systolic Blood Pressure cat [3] | 0.887 | 0.748 – 1.054 | 0.171 |
| Min 24 Hour Systolic Blood Pressure cat [4] | 1.088 | 0.579 – 1.926 | 0.781 |
| poscx012 cl [1] | 7.512 | 6.501 – 8.693 | **<0.001** |
| Observations | 15704 | | |
| R^2^ Tjur | 0.146 | | |

**2c. Adjusted analyses treating quarter as a continuous variable, COVID-19 with Sepsis, Excludes Quarter 1**

|  | **Prolonged Antibiotics** | | |
| --- | --- | --- | --- |
| *Predictors* | *Odds Ratios* | *95% CI* | *p* |
| (Intercept) | 0.118 | 0.029 – 0.425 | **0.002** |
| Quarter [categorical, Ref=1] | 1.023 | 0.992 – 1.054 | 0.147 |
| Encounter age [continuous] | 1.000 | 0.994 – 1.006 | 0.972 |
| sex [Male] [binary, Ref=female] | 1.171 | 1.001 – 1.370 | **0.048** |
| Hispanic, all races [categorical, Ref=Non-Hispanic White] | 0.748 | 0.591 – 0.947 | **0.016** |
| Non-Hispanic Black | 0.802 | 0.611 – 1.050 | 0.110 |
| Non-Hispanic Other, including Asian and Multi-racial | 0.718 | 0.524 – 0.981 | **0.038** |
| Hospital Site 2 [categorical, Ref= Hospital Site 1] | 1.159 | 0.854 – 1.569 | 0.342 |
| Hospital Site 3 | 1.167 | 0.957 – 1.424 | 0.127 |
| Hospital Site 4 | 0.754 | 0.582 – 0.974 | **0.031** |
| Hospital Site 5 | 0.650 | 0.510 – 0.827 | **<0.001** |
| insurancetype [Medicaid] [categorical, Ref=private] | 1.100 | 0.853 – 1.418 | 0.461 |
| insurancetype [Medicare] | 0.963 | 0.804 – 1.152 | 0.679 |
| BMI <18.5 [categorical, Ref=BMI 18.5-24.9] | 1.713 | 1.148 – 2.569 | **0.009** |
| BMI >=25 & <30 | 0.957 | 0.780 – 1.173 | 0.671 |
| BMI>=30 & <35 | 0.948 | 0.751 – 1.197 | 0.655 |
| BMI >=35 | 0.872 | 0.689 – 1.103 | 0.253 |
| Elixhauser index mortality [continuous] | 1.008 | 1.002 – 1.014 | **0.010** |
| Elixhauser cancer [binary] | 1.203 | 0.940 – 1.539 | 0.142 |
| Elixhauser diabetes [binary] | 1.172 | 0.990 – 1.389 | 0.066 |
| Elixhauser lung chronic [binary] | 1.273 | 1.073 – 1.509 | **0.006** |
| Elixhauser heart failure [binary] | 1.035 | 0.840 – 1.274 | 0.749 |
| Elixhauser hypertension [binary] | 0.925 | 0.763 – 1.121 | 0.426 |
| Elixhauser renal failure [binary] | 0.957 | 0.787 – 1.164 | 0.661 |
| Elixhauser liver disease [binary] | 1.427 | 1.094 – 1.863 | **0.009** |
| Max 24 Hour temp >=96.8 & <100.4 [categorical, REF= <96.8] | 3.824 | 1.365 – 12.627 | **0.016** |
| Max 24 Hour temp >=100.4 | 5.977 | 2.114 – 19.866 | **0.001** |
| Max 24 Hour Heart Rate >=91 & <121 [categorical, REF= >=41 &<91] | 1.072 | 0.877 – 1.311 | 0.497 |
| Max 24 Hour Heart Rate >=121 | 1.016 | 0.796 – 1.296 | 0.899 |
| Max 24 Hour Respiratory Rate >=21 & <31 [categorical, REF=>=11 & <21] | 1.340 | 1.033 – 1.743 | **0.028** |
| Max 24 Hour Respiratory Rate >=31 | 1.503 | 1.137 – 1.991 | **0.004** |
| Max 24 Hour lactate >=2 & <3 [categorical, REF= <2] | 0.871 | 0.698 – 1.088 | 0.224 |
| Max 24 Hour lactate >=3 & <4 | 1.237 | 0.903 – 1.697 | 0.186 |
| Max 24 Hour lactate cat >=4 | 0.926 | 0.697 – 1.232 | 0.599 |
| Max 24 Hour lactate missing/lactate not obtained | 0.561 | 0.459 – 0.686 | **<0.001** |
| Max 24 Hour Oxygen Device: nasal cannula [categorical, REF= none] | 0.965 | 0.546 – 1.763 | 0.905 |
| Max 24 Hour Oxygen Device: simple mask | 1.140 | 0.634 – 2.118 | 0.669 |
| Max 24 Hour Oxygen Device: oxymizer | 0.944 | 0.436 – 2.055 | 0.883 |
| Max 24 Hour Oxygen Device: advanced mask | 0.921 | 0.509 – 1.719 | 0.790 |
| Max 24 Hour Oxygen Device: high flow nasal canula | 1.560 | 0.858 – 2.926 | 0.154 |
| Max 24 Hour Oxygen Device: BIPAP | 1.303 | 0.606 – 2.838 | 0.501 |
| Max 24 Hour Oxygen Device: ventilator | 1.391 | 0.774 – 2.577 | 0.281 |
| Max 24 Hour Oxygen Device: ECMO | 115499.025 | 0.000 – NA | 0.953 |
| Min 24 Hour Systolic Blood Pressure >=90 & <121 [categorical, REF=<=89] | 0.663 | 0.549 – 0.800 | **<0.001** |
| Min 24 Hour Systolic Blood Pressure >=121 & <161 | 0.540 | 0.408 – 0.713 | **<0.001** |
| Min 24 Hour Systolic Blood Pressure cat >=161 | 0.666 | 0.133 – 2.697 | 0.583 |
| positive culture (any site) before HD 3 [binary] | 4.245 | 3.342 – 5.430 | **<0.001** |
| Observations | 3339 | | |
| R^2^ Tjur | 0.169 | | |

**2d. Adjusted analyses treating quarter as a continuous variable, COVID-19 without Sepsis, excludes Quarter 1**

|  | **Prolonged Antibiotics** | | |
| --- | --- | --- | --- |
| *Predictors* | *Odds Ratios* | *95% CI* | *p* |
| (Intercept) | 0.133 | 0.030 – 0.409 | **0.002** |
| Quarter [categorical, Ref=1] | 1.014 | 0.996 – 1.032 | 0.118 |
| Encounter age [continuous] | 0.997 | 0.994 – 1.001 | 0.123 |
| sex [Male] [binary, Ref=female] | 1.318 | 1.195 – 1.453 | **<0.001** |
| Hispanic, all races [categorical, Ref=Non-Hispanic White] | 0.706 | 0.609 – 0.818 | **<0.001** |
| Non-Hispanic Black | 0.754 | 0.637 – 0.889 | **0.001** |
| Non-Hispanic Other, including Asian and Multi-racial | 0.634 | 0.513 – 0.778 | **<0.001** |
| Hospital Site 2 [categorical, Ref= Hospital Site 1] | 1.070 | 0.902 – 1.266 | 0.434 |
| Hospital Site 3 | 1.162 | 1.020 – 1.323 | **0.023** |
| Hospital Site 4 | 0.714 | 0.605 – 0.839 | **<0.001** |
| Hospital Site 5 | 0.958 | 0.832 – 1.103 | 0.551 |
| insurancetype [Medicaid] [categorical, Ref=private] | 1.071 | 0.921 – 1.243 | 0.371 |
| insurancetype [Medicare] | 1.004 | 0.893 – 1.127 | 0.953 |
| BMI <18.5 [categorical, Ref=BMI 18.5-24.9] | 1.386 | 1.079 – 1.771 | **0.010** |
| BMI >=25 & <30 | 0.784 | 0.692 – 0.887 | **<0.001** |
| BMI>=30 & <35 | 0.663 | 0.570 – 0.770 | **<0.001** |
| BMI >=35 | 0.636 | 0.551 – 0.733 | **<0.001** |
| Elixhauser index mortality [continuous] | 1.003 | 0.999 – 1.007 | 0.145 |
| Elixhauser cancer [binary] | 1.793 | 1.537 – 2.090 | **<0.001** |
| Elixhauser diabetes [binary] | 1.159 | 1.035 – 1.298 | **0.011** |
| Elixhauser lung chronic [binary] | 1.287 | 1.150 – 1.438 | **<0.001** |
| Elixhauser heart failure [binary] | 1.057 | 0.908 – 1.229 | 0.472 |
| Elixhauser hypertension [binary] | 0.961 | 0.850 – 1.086 | 0.521 |
| Elixhauser renal failure [binary] | 1.094 | 0.961 – 1.246 | 0.174 |
| Elixhauser liver disease [binary] | 1.410 | 1.190 – 1.666 | **<0.001** |
| Max 24 Hour temp >=96.8 & <100.4 [categorical, REF= <96.8] | 1.832 | 0.626 – 7.897 | 0.334 |
| Max 24 Hour temp >=100.4 | 2.850 | 0.969 – 12.325 | 0.096 |
| Max 24 Hour Heart Rate >=91 & <121 [categorical, REF= >=41 &<91] | 1.058 | 0.950 – 1.179 | 0.306 |
| Max 24 Hour Heart Rate >=121 | 1.032 | 0.874 – 1.217 | 0.706 |
| Max 24 Hour Respiratory Rate >=21 & <31 [categorical, REF=>=11 & <21] | 0.958 | 0.858 – 1.069 | 0.442 |
| Max 24 Hour Respiratory Rate >=31 | 1.025 | 0.868 – 1.207 | 0.772 |
| Max 24 Hour lactate >=2 & <3 [categorical, REF= <2] | 1.218 | 0.980 – 1.510 | 0.073 |
| Max 24 Hour lactate >=3 & <4 | 1.500 | 1.062 – 2.101 | **0.020** |
| Max 24 Hour lactate cat >=4 | 1.355 | 0.926 – 1.961 | 0.112 |
| Max 24 Hour lactate missing/lactate not obtained | 0.544 | 0.489 – 0.605 | **<0.001** |
| Max 24 Hour Oxygen Device: nasal cannula [categorical, REF= none] | 1.098 | 0.982 – 1.226 | 0.101 |
| Max 24 Hour Oxygen Device: simple mask | 3.237 | 0.998 – 10.828 | **0.050** |
| Max 24 Hour Oxygen Device: oxymizer | 0.000 | NA – 6094966089435.234 | 0.957 |
| Max 24 Hour Oxygen Device: advanced mask | 386381.830 | 0.000 – NA | 0.948 |
| Max 24 Hour Oxygen Device: high flow nasal canula | 1.067 | 0.053 – 7.858 | 0.955 |
| Max 24 Hour Oxygen Device: BIPAP | 3.445 | 0.110 – 108.200 | 0.436 |
| Max 24 Hour Oxygen Device: ventilator | 0.000 | NA – 13564024706261.348 | 0.960 |
| Min 24 Hour Systolic Blood Pressure >=90 & <121 [categorical, REF=<=89] | 1.012 | 0.858 – 1.196 | 0.892 |
| Min 24 Hour Systolic Blood Pressure >=121 & <161 | 0.885 | 0.733 – 1.070 | 0.204 |
| Min 24 Hour Systolic Blood Pressure cat >=161 | 0.985 | 0.477 – 1.860 | 0.965 |
| positive culture (any site) before HD 3 [binary] | 8.139 | 6.956 – 9.538 | **<0.001** |
| Observations | 13470 | | |
| R^2^ Tjur | 0.149 | | |

**Supplement Table 3. Summary of results of models for receipt of any initial antibiotics**

|  | | **With Sepsis** | **Without Sepsis** |
| --- | --- | --- | --- |
| **Entire Study Period (Q1-11)** | | | |
|  | Crude model, IRR (95%CI) | 0.976 (0.965-0.986)* | 0.972 (0.965-0.980)* |
|  | Adjusted model, OR (95%CI) | 0.932 (0.910-0.955)* | 0.968 (0.956-0.979)* |
| **Excluding Quarter 1 (Q2-11)** | | | |
|  | Crude model, IRR (95%CI) | 1.016 (1.000-1.033) | 1.008 (0.997-1.019) |
|  | Adjusted model, OR (95%CI) | 1.030 (0.997-1.063) | 1.015 (1.000-1.031) |

***Indicates statistical significance p<0.001**

**Supplement Figure 1. Adjusted Rates of Initial Antibiotics in Hospitalized COVID-19 Patients Presenting With (A) vs. Without (B) Signs of Sepsis**  **
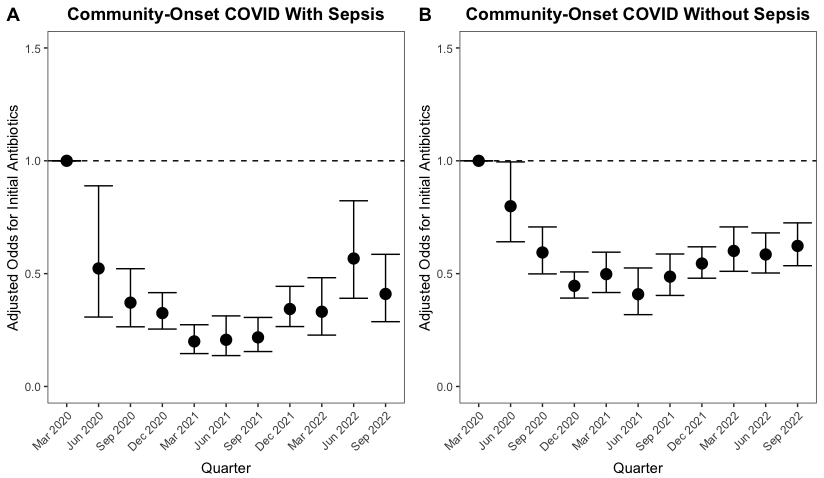
**

**Supplement Figure 2. Crude and Adjusted Rates of Early (hospital day -1 to 2) Microbiologic Culture including Blood, Sputum, or Urine in Hospitalized COVID-19 Patients Presenting With vs Without Signs of Sepsis**


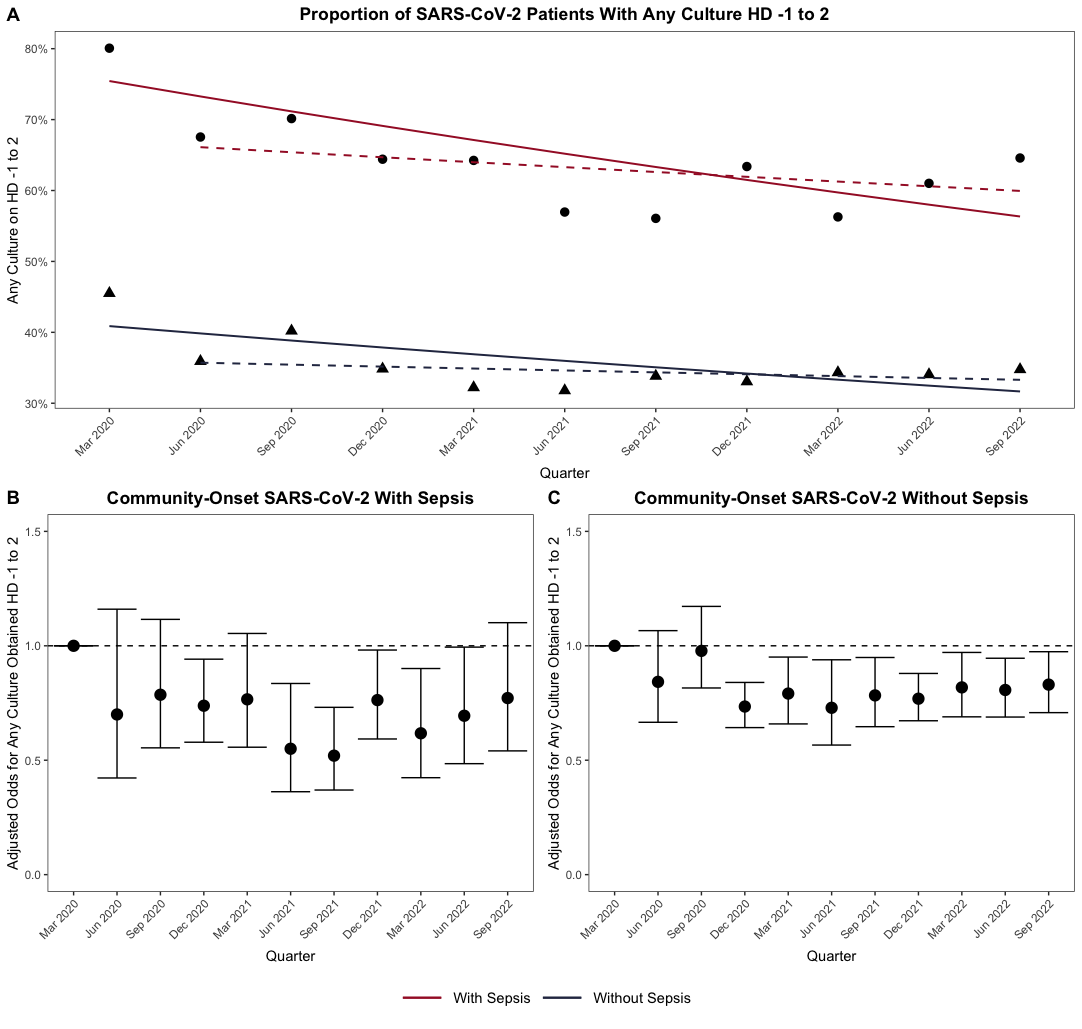

Supplement: Shappell et al. supplementary material [file S2732494X24003668sup001.docx]
